# Biogeochemical Cycle of Methanol in Anoxic Deep-Sea Sediments

**DOI:** 10.1264/jsme2.ME15204

**Published:** 2016-06-10

**Authors:** Katsunori Yanagawa, Atsushi Tani, Naoya Yamamoto, Akihiro Hachikubo, Akihiro Kano, Ryo Matsumoto, Yohey Suzuki

**Affiliations:** 1 Faculty of Social and Cultural Studies, Kyushu UniversityFukuoka 819–0395Japan; 2 Department of Earth and Space Science, Graduate School of Science, Osaka UniversityToyonaka, Osaka, 560–0043Japan; 3 Environmental and Energy Resources Research Center, Kitami Institute of TechnologyKitami 090–8507Japan; 4 Gas Hydrate Laboratory, Organization for the Strategic Coordination of Research and Intellectual Properties, Meiji UniversityChiyoda-ku, Tokyo 101–8301Japan; 5 Department of Earth and Planetary Science, Graduate School of Science, University of TokyoBunkyo-ku, Tokyo, 113–0033Japan

**Keywords:** methanol consumption, methanol production, marine sediment

## Abstract

The biological flux and lifetime of methanol in anoxic marine sediments are largely unknown. We herein reported, for the first time, quantitative methanol removal rates in subsurface sediments. Anaerobic incubation experiments with radiotracers showed high rates of microbial methanol consumption. Notably, methanol oxidation to CO_2_ surpassed methanol assimilation and methanogenesis from CO_2_/H_2_ and methanol. Nevertheless, a significant decrease in methanol was not observed after the incubation, and this was attributed to the microbial production of methanol in parallel with its consumption. These results suggest that microbial reactions play an important role in the sources and sinks of methanol in subseafloor sediments.

Among volatile organic compounds, methanol is considered the most attractive option for investigating global biogeochemical cycling. Methanol is produced during the anaerobic decomposition of organic matter (19) and is consumed by methylotrophic bacteria for aerobic respiration (1–3, 13, 17). Several studies have demonstrated that methanol is utilized biologically as carbon and energy sources in the ocean (6, 9, 20), resulting in the formation of a considerable carbon reservoir (10). Furthermore, methanol is known to degrade in anoxic environments in association with denitrification (11), iron reduction (5), sulfate reduction (16), and methanogenesis (4, 12, 24, 27). Among these anaerobic methanol oxidation reactions, methylotrophic methanogenesis is particularly notable because methylotrophic methanogens are not outcompeted by sulfate reducers in sulfate-rich environments (18). However, limited information is currently available on the quantitative distribution of methanol under anoxic sedimentary conditions because of its low concentration and high solubility in pore water. Only one previous study is known to have shown micromolar levels of methanol in shallow marine sediments in the Black Sea and Gulf of Mexico (28). However, methanol concentrations at deeper depths and the turnover rates of methanol in deep-sea sediments have not yet been investigated. Therefore, we herein examined the microbial consumption of methanol in deep-sea sediments from the Umitaka Spur in the eastern Japan Sea.

Sediment cores were collected using a giant piston corer (Calypso) during the MD179 expedition with the R/V Marion Dufresne in June, 2010. The two sediment cores (MD3296: 37°24.810 E, 138°00.800 E and MD3301: 37°27.590N, 138°04.600E), collected several kilometers from a gas seep site were cut into 1.5-m sections immediately after retrieval. Pore water and sediment samples for geochemical and microbiological studies were collected as previously described (8, 26). Methanol concentrations were measured by gas chromatography coupled with mass spectrometry (Clarus 600 GC-MS, PerkinElmer, Waltham, MA, USA) as described elsewhere (25).

Methanol was maintained at low concentrations of 0.3–3.2 μM in shallow sediments above the sulfate-methane transition zone (SMTZ; approximately 5 m below the seafloor [mbsf] of MD3301 and approximately 3 mbsf of MD3304) (Fig. 1). However, the concentration of methanol began to increase gradually from below the SMTZ to approximately 20 μM near the bottom of the core at approximately 30 mbsf. The profiles of methanol concentrations suggest that *in situ* methanol production exceeds methanol consumption and/or that methanol diffuses from any deep source. The concentration of methanol abruptly decreased in the lowermost part of the MD3304 core. Although the reasons for this decrease currently remain unclear, similar geochemical demarcation was observed in the profile of Cl (22). These subseafloor methanol profiles are wider than previously reported intervals. Additionally, the concentration range in the Japan Sea is higher than in the Black Sea, but lower than in the Gulf of Mexico (28).

The methanol removal rate was determined from onshore incubation experiments using sediment slurry samples collected at different depths. Sediment samples were anaerobically stored at 4°C in glass vials in which the headspace gas was replaced by argon immediately after sampling. One milliliter of stored sediment samples was amended with 5 mL of anoxic artificial seawater to prepare slurry samples for radiotracer experiments. The incubation was performed at 4°C with ^14^C-labeled substrates (American Radiolabeled Chemicals, Saint Louis, MO, USA) for 50 d in the radiation controlled area of the Japan Agency for Marine-Earth Science and Technology (JAMSTEC) at Yokosuka, Kanagawa, Japan. The radioactivity of ^14^C-methanol was 75 kBq, and the initial total concentration of methanol (including the concentrations of the original sediment, radiotracer, and artificial seawater) was designed to be approximately 40 μM. The potential rates of anaerobic methanol consumption (methanol oxidation to CO_2_, methanogenesis from methanol, and methanol assimilation into particulate cellular material) were determined based on the radioactivity of ^14^C-methanol-derived products. The rates of hydrogenotrophic methanogenesis were also estimated from the rate of conversion from ^14^C-bicarbonate to ^14^CH_4_ as reference microbial activity (21). The radioactivities of microbially produced ^14^CO_2_ and ^14^CH_4_ in the headspace were measured using a gas chromatograph (Shimadzu GC-2014, Shimadzu, Kyoto, Japan) and highly sensitive radioactivity detector (RAGA Star, Raytest, Straubenhart, Germany). The rates of methanol assimilation were determined from the amount of particulate cellular material that was newly synthesized from ^14^C-methanol. The radioactivity of ^14^C-incorporated cells on a 0.2-μm pore polycarbonate filter (Merck Millipore, Darmstadt, Germany) was determined using a liquid scintillation counter (Tri-Carb 2900TR, PerkinElmer). Potential activities were calculated based on the proportion of the radioactive ^14^C-product to the total radioactive substrate, the concentrations of methanol and bicarbonate, and the incubation time. Although methanol is utilized as a substrate for methylotrophic methanogenesis and sulfate reduction in anaerobic environments, our radiotracer experiments demonstrated that methanol oxidation activities outcompeted methanogenesis from methanol, and were sustained under low sulfate conditions below the SMTZ (Fig. 1). One plausible explanation for this is that methanol was converted to acetate via organoheterotrophic acetogenesis (15), which was finally oxidized to CO_2_ as the end product. Anaerobic methanol oxidation activities were one to two orders of magnitude higher than those of methanol assimilation (Fig. 1), indicating that more abundant microbes used methanol as an energy source through dissimilation to CO_2_ than as a carbon source via assimilation.

The biological turnover of methanol suggested that methanol in the sediment samples may disappear within a few months (Fig. 1). However, no significant loss of methanol was observed after three-month incubation experiments, which were conducted in parallel with the radiotracer experiments (Fig. 2A). This may have been due to the generation of methanol in the sediment samples. The potential rates of methanol production and consumption were calculated based on the measured concentration change and radiotracer experiments, respectively (Fig. 2B). The methanol produced is interpreted as a metabolic intermediate during the microbial degradation of organic matter, such as lignin, pectin, and carbohydrates, under anoxic conditions (7, 13, 19). These microbial activities may supply a higher amount of methanol in deep sediments, which may further induce a high consumption rate of methanol and lead to the high replacement of methanol at the same depth.

The results of the present study revealed the depth profiles and rapid turnover of methanol in marine subsurface sediments in the eastern Japan Sea. The methanol profiles and potential production rates obtained suggest that methanol production is regulated by the state of the diagenesis of organic compounds in the sediment. Our results also indicate that the balance between *in situ* methanol production and consumption by subseafloor microbial populations is close to a state of dynamic equilibrium. Methanol depth profiles in marine sediments may be controlled by a production-consumption imbalance of methanol, as observed in the Black Sea and Gulf of Mexico (28). In the present study, methanol profiles and potential removal rates differed slightly between cores MD3301 and MD3304, despite a short separation distance of only 7 km. Although subseafloor microbial cell abundance, which was evaluated using SYBR Green I as described previously (23), did not differ significantly between sites, the entire microbial community structure changed slightly (Fig. 1). This may have resulted from site-to-site variations in the diagenesis of organic matter in the sediments. Organic matter diagenesis may also affect the production of methanol and abundance and activity of microbial populations responsible for methanol consumption.

We may have overlooked the importance of methanol to microbial community development in marine subsurface sediments. It is also necessary to clarify methanol biogeochemical cycles in anoxic terrestrial environments because methanol is a wood alcohol and product of terrestrial plants. Future studies need to focus on the microbial population responsible for methanol biogeochemical cycles in anoxic environments. The contribution of anaerobic methanol utilizers, including methylotrophic methanogens and acetogens, will be clarified from specific gene analyses on homologs of methanol dehydrogenase, methanol oxidoreductase, methanol oxidase, and methanol: corrinoid methyltransferase in anaerobic marine sediments (14).

## Figures and Tables

**Fig. 1:**
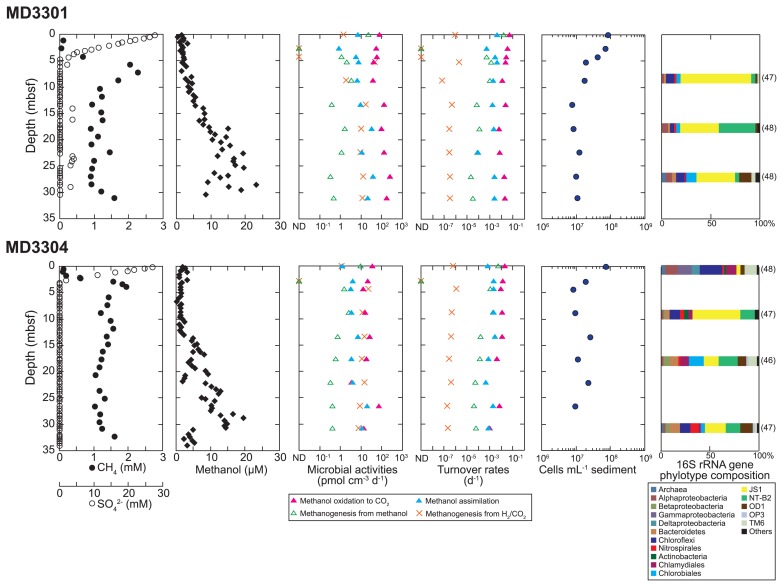
Depth profiles of pore water geochemistry, potential microbial activities, turnover rates, numbers of microbial cells, and prokaryotic 16S rRNA gene phylotype compositions in MD3301 and 3304 cores. The values below the detection limit for microbial activities and the turnover rates are plotted on the left axes. The relative abundances of phylum/class-level phylotypes are shown in each column diagram in the left graph. The numbers in parentheses indicate the number of phylotypes. The concentrations of methane and sulfate and the microbial community compositions were originally reported elsewhere (26).

**Fig. 2:**
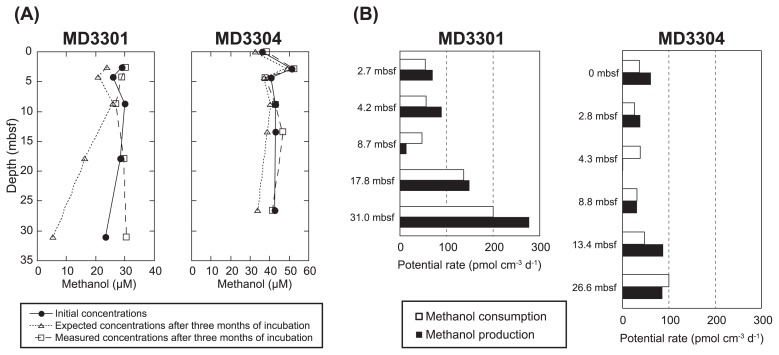
Potential rates of methanol production and consumption. (A) Absolute concentrations in pore water methanol before and after a three-month incubation. The changes measured in methanol concentration were higher than those expected from the sum of microbial methanol consumption activities (anaerobic methanol oxidation, methanol assimilation, and methanogenesis from methanol), which were determined based on the radiotracer experiments in Fig. 1. (B) Comparison of potential methanol consumption rates and production rates. Potential methanol production rates were estimated from the difference between the expected values from methanol consumption activities and the changes measured in methanol concentration after the incubation.
